# Dental Furnishing Establishments

**Published:** 1853-01

**Authors:** 


### DENTAL FURNISHING ESTABLISHMENTS.

Next in importance to a thorough education in Dentistry, is good materials and facilities for obtaining them. The largest assortment, and greatest variety (comprising everything needed in the practice of Dentistry) to be found in any *one* establishment either east or west, is kept by J. M. Brown, cor. Fourth and Walnut streets, Cincinnati.

We are pleased to see an increased stock of new teeth of the most beautiful shapes and colors, the prices of which have been reduced to the same rates charged by the Eastern manufacturers.

The present express and mail facilities, added to the uniform promptness with which business is dispatched at this establishment, gives the profession in the West and South, unequalled advantages in *price* and *time*.

Every article is warranted satisfactory or the money refunded.

